# Application and design of esterase-responsive nanoparticles for cancer therapy

**DOI:** 10.1080/10717544.2019.1588424

**Published:** 2019-03-31

**Authors:** Haonan Dong, Long Pang, Hailin Cong, Youqing Shen, Bing Yu

**Affiliations:** a Institute of Biomedical Materials and Engineering, College of Chemistry and Chemical Engineering, College of Materials Science and Engineering, Qingdao University, Qingdao, Shandong, P.R. China;; b State Key Laboratory of Bio-Fibers and Eco-Textiles, Qingdao University, Qingdao, Shandong, P.R. China;; c Key Laboratory of Biomass Chemical Engineering of Ministry of Education, Center for Bionanoengineering, and Department of Chemical and Biological Engineering, Zhejiang University, Hangzhou, Zhejiang, P.R. China

**Keywords:** Esterase-responsive nanoparticles, drug carriers, gene therapy, photosensitizers, fluorescent imaging

## Abstract

Nanoparticles have been developed for tumor treatment due to the enhanced permeability and retention effects. However, lack of specific cancer cells selectivity results in low delivery efficiency and undesired side effects. In that case, the stimuli-responsive nanoparticles system designed for the specific structure and physicochemical properties of tumors have attracted more and more attention of researchers. Esterase-responsive nanoparticle system is widely used due to the overexpressed esterase in tumor cells. For a rational designed esterase-responsive nanoparticle, ester bonds and nanoparticle structures are the key characters. In this review, we overviewed the design of esterase-responsive nanoparticles, including ester bonds design and nano-structure design, and analyzed the fitness of each design for different application. In the end, the outlook of esterase-responsive nanoparticle is looking forward.

## 1. Introduction

Nowadays, cancer has become one of the most ruthless killers of human, leading to more than 8 million deaths annually all over the world (Zhou et al., 2018). In addition to surgery, radiation therapy, and immunotherapy, as another major treatment for cancer, chemotherapeutic drug therapy is an important method for clinical treatment of cancer. Most of the anticancer drugs, however, destroy cancer cells as well as healthy tissues due to lack of tumor-selectivity. How to delivery anticancer drug into tumor cells is the key problem in cancer therapy. Cancer nanomedicines have been designed for delivering drugs to tumor tissues by exploting tumor’s enhanced permeability and retention effect that greatly improved the efficiency of drugs (Maeda, 2015). The studies of drug nanodelivery have been developed explosively in recent years. With the development of nanodelivery, gene plasmids (Wu et al., 2017), photosensitizers (Hou et al., 2018), and imaging agents (Zhang & Zhao, 2014) are also transported by nanocarriers for tumor therapy or imaging.

**Figure 1.**
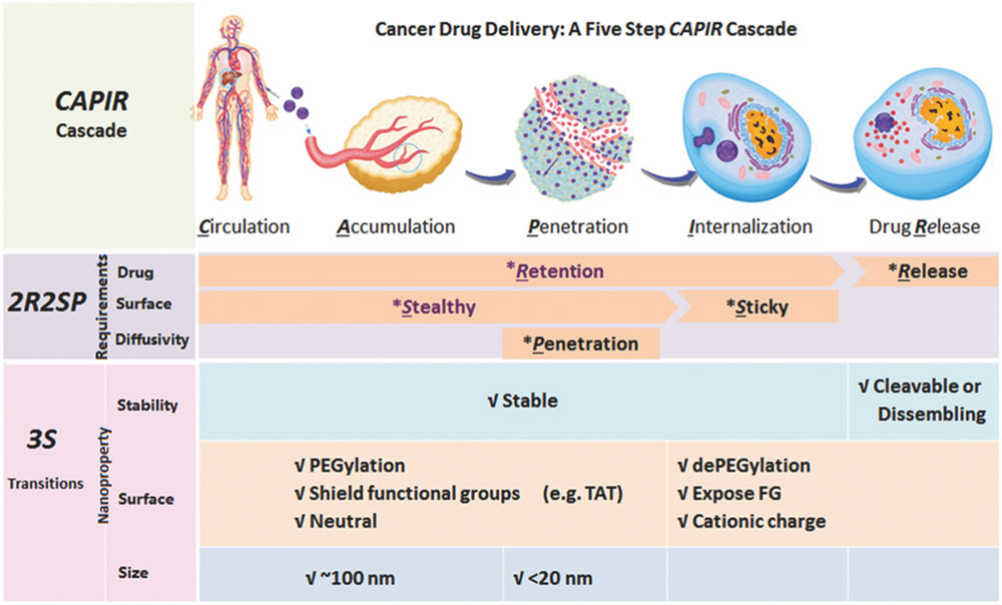
CAPIR cascades for cancer drug delivery (Sun et al., 2017). Copyright 2017, Wiley.

For a nanomedicine, it is not an easy process to deliver drugs to tumor tissue. According to Sun et al. (2017)’s study, to delivery nanomedicine into tumor cells, five steps must be gone through: circulation, accumulation, penetration, internalization, and release (CAPIR cascade). The ‘passengers’ should stay in the ‘nano-bus’ among the CAPI steps and get off at the R step, which is called stability transition (Figure 1). In the past decades, by utilizing microenvironment difference between tumor tissues and normal tissues, such as pH, reactive oxygen species (ROS), glutathione (GSH), and overexpressed enzyme, numerous stimulus-response nanoparticles were designed to achieve the stability transitions (Chen et al., 2017). Esterase is one of the overexpressed enzymes in tumor cells (Wells & Grandis, 2003). For example, esterase activity is around 2.6- to 3.7-fold in malignant colorectal tumor (0.45 ± 0.25 U/L for male and 0.45 ± 0.35 U/L for female) than in normal tissues (0.17 ± 0.09 U/L for male and 012 ± 0.07 U/L for female) (Niu et al., 2012). In cancer cell lines, the overexpressed esterase plays an important role in migration, invasion, survival, and *in vivo* tumor growth. Studies have shown that stored fats in cancer cells are liberated to support cancer pathogenesis under esterase catalysis (Nomura et al., 2010; Qin & Ruan, 2014). Utilizing the high level of esterase in tumor cells, esterase-responsive nanoparticles could reveal stability transition in the R step and a lot of esterase-responsive nanoparticles have been designed.

Herein, we analysis nanoparticles designed to achieve the stability transitions with esterase-response type. Moreover, the cargoes delivered by nanoparticles in this review are not only chemotherapy drugs. Gene plasmids, photosensitizer [for photodynamic therapy (PDT), photothermal therapy (PTT), etc.], and imaging agents are also overviewed.

## 2. Specific enzymes in tumor tissues

As a natural biocatalyst, enzymes are not only the necessary regulatory substances for metabolism of living organisms, but also the biological basis for the stimuli-responsive release that nanoparticles can utilize. Cells contain a variety of enzymes, such as esterase (Kashima et al., 2000; Camacho et al., 2009), lipase (Camacho et al., 2009), phospholipase (Dennis et al., 2011), nuclease (Elkon, 2018), and the like. Linking the enzymatically sensitive chemical bonds between drugs and carriers triggers the controlled release of drugs under the catalysis of the enzyme (He et al., 2016). Especially in the tumor tissues, the content of various specific enzymes, such as matrix metalloproteinases (MMP) (Leeman et al., 2003), phospholipase (Qiu et al., 2016), esterase (Hatakeyama et al., 2011), are much larger than the other normal tissues. This can be used to achieve selective release of cargoes, like genes and drugs, etc. For example, short peptides that are sensitive to proteases can be conjugated to polyethylene glycol (PEG) and the PEG shell can be split off to expose the drug core (Bernardos et al., 2010; Zhu et al., 2012). The grafting of the polysaccharide onto silica can be broken to release drugs under the action of lysosomal enzymes (Carr & Jiang, 2010).

Esterase belongs to the class of hydrolases and is widely present in the cytoplasm and nucleus of humans and animals. The esterase-sensitive chemical bond such as carboxylate ester bond is introduced into the structure of the carrier, and the ester bond can be hydrolyzed under the intracellular esterase to break the stable structure between drugs and carriers or to activate photosensitizer. This hydrolysis process is very significant, and it can be realized that the carrier can be combined with antineoplastic agents in the CPIR steps and can be rapidly hydrolyzed by an esterase to release antineoplastic agents. However, some ester structures, such as alkyl esters (Qiu et al., 2018), hydrolyze very slowly, even in the presence of esterase. Therefore, rational design of esterase-responsive nanoparticles is needed to efficiently delivery drugs into tumor cells.

## 3. Design of esterase-responsive nanoparticles

### 3.1. Ester bonds design

Due to the great differences in esterase responsiveness of compounds with different ester bond structures, nanoparticles should be designed with a proper ester bond as required. Therefore, to realize smart-release goals (such as rapid release, accurate release or selective release), several strategies of rationally designed ester bonds are introduced below.

For most of drug or gene plasmid, rapid response to esterase is required to induce apoptosis of tumor cells within a short time. Phenyl hydroxyl ester is much more reactive than alkyl ester based on extensive literature research (Qiu et al., 2016, 2018). Phenyl hydroxyl esters have been utilized most commonly for rapid esterase-responsive nanocarriers’ design. As several anticancer drugs possess phenyl hydroxyl groups, they could be esterified to form prodrugs with rapid esterase-responsive property. 10-hydroxycamptothecin (HCPT), a most common anticancer chemotherapy drug, for instance, possess two kinds of hydroxyl groups, an alkyl hydroxyl group (20-hydroxyl) and a phenyl hydroxyl group (10-hydroxyl). The 20-ester of HCPT is more unstable than the 10-ester under esterase catalysis (He et al., 2016). The results showed that alkyl esters show much lower hydrolysis activity than phenyl hydroxy esters. Besides phenyl esters, some other unique ester groups are synthesized to meet the requirement of rapid esterase-responsive nanoparticles. Carbonic esters structure is regarded as one of the rapid response groups of esterase. Once a small amount carbonic ester group was introduced into the polycaprolactone (PCL) molecular chain, its hydrolysis rate with esterase was much faster than pure PCL (Yasuda et al., 1999). According to this phenomenon, carbonated PCL is regarded as a suitable substitute for pure PCL in drug delivery. Carbonic esters are also used to link drugs and carriers to form prodrugs, that drugs would release rapidly when exposed to intracellular esterase in cancer cells (Han et al., 2017; Liu et al., 2017; Sadrerafi et al., 2018). Another labile ester group is called alkanoyloxymethyl ester. The most commonly alkanoyloxymethyl ester is acetoxymethyl ester that can be readily hydrolyzed by the esterase in tumor cells. For example, Sakai et al. (2017) synthesized a series of alkanoyloxymethyl esters to inactivate terpyridine, a europium (III) ligand, for measuring tumor cell cytotoxicity mediated by immune cells. These proligands have been proved an explosive release of terpyridine when labeling tumor cells due to the high intracellular esterase concentration. Several esterase-responsive prodrugs based on alkanoyloxymethyl ester have been designed (Burks et al., 2008; Matsumoto et al., 2016; Tanaka et al., 2017). The rapid esterase-responsive bonds were shown in Table 1. Combination of these rapid hydrolysis ester groups would accelerate the hydrolysis rate of esters, however, also make the esters bond unstable inevitably (He et al., 2004). The unstable ester bonds, on the contrary, would fail to meet the requirement for drug delivery. A 10-carbonate of HCPT could hydrolysis in human plasma, and as a result, to release drugs. The drug could not arrive at their destination and will have toxic effects on normal tissues. Therefore, according to the practical requirement, the ester structure of nanoparticles should be rationally designed.

**Table 1.** Rapid esterase-responsive bonds strategy.

Structure of rapid esterase-responsive bonds	Reference
	He et al. (2016) Han et al. (2017); Liu et al. (2017)25–27; Sadrerafi et al. (2018) Burks et al. (2008)29–31; Matsumoto et al. (2016); Tanaka et al. (2017)

Another strategy to realize esterase responsiveness is that esterification of carboxylic acid and unstable hydroxyl group to form a prodrug. These kinds of prodrugs could be delivered to tumor cells and supply antitumor drugs or imaging agents by ester bonds hydrolysis to carboxylic acid and unstable alcohol or phenol, which is further transformed into active form. For example, Zheng et al. (2017) have designed a prodrug with ester of hydroxymethyl disulfide structure, similar to the idea of hemiacetal. The prodrug is stable under the protection of ester bond at physiological conditions. Once triggered by esterase, the prodrug could be divided into a molecule of carboxylic acid and a molecule of hydroxymethyl disulfide analog, an unstable intermediate would further release hydrogen sulfide under high GSH concentration. Ji et al. (2017), in a different method, provided a prodrug of carbon monoxide (CO) with esterase sensitivity. A 7-members ring with an ester bond is utilized to hold the alkyne group from the dienone moiety. Once in the present of esterase, the 7-members ring is hydrolyzed and the alkyne group is freed for cycloaddition to release CO. Except inorganic molecules, organic chemotherapeutics drugs can also form prodrug with this strategy. Ma et al. (2015) utilized esterification of catechol of β-lapachone to form two kind of prodrugs with high loading content property in PEG-b-PLA micelles. These prodrugs could be hydrolysed under high intercellular esterase concentration of tumor cells and intramolecular structure rearranged into benzoquinone structure, which is the activated form to induce tumor cells apoptosis. Similar as β-lapachone, fluorescein (Pang et al., 2017) also possesses phenol hydroxyl structure or benzoquinone structure, which is nonfluorescent or fluorescent, respectively. Fluorescein diacetate, which is fixed to the phenol structure by ester bond, has become the most commonly used probe for detecting esterase concentration in tumor cells (Nilewski et al., 2018). Kusaka et al. (2013) masked the hydroxy group on coumaric acids with an ester bond, which then are conjugated to gold nanorod. The ester bond could be cleaved by esterase and at the same time, form lactone from coumarins that have fluorescent property. Moreover, *p*-alkylacyloxyl benzyl alcohol is easily hydrolysed under esterase condition (Perez et al., 2013). Once the phenol hydroxy groups exposed, prodrug with this structure undergo a rapid electron transfer, followed by the release of *p*-quinone methide that quickly turns into *p*-hydroxymethyl phenol in water. Perez et al. (2013) detailed the comprehensive evaluation of strategies for the effective release of hydroxyl and phenolic moieties in the presence of esterase. They used matrix MMPs as a concept target. Three different ester reactive protecting groups were incorporated into the MMP preinhibitor containing the hydroxyl moiety. Analytical evaluation of preinhibitors showed that the use of the *p*-acetoxyl benzyl ether protecting group resulted in rather rapid conversion kinetics and enhanced the water stability compared to the more conventional method where the trigger was directly linked to the inhibitor. Some examples of this strategy were shown in Table 2. This strategy meets the stable transition requirement greatly and will rapidly develop in antitumor section.

**Table 2.** Examples of ‘masking’ strategies for esterase-responsive bonds.

Prodrugs Intermediates Stable compounds	Reference
	Zheng et al. (2017) Ji et al. (2017) Nilewski et al. (2018) Perez et al. (2013)

### 3.2. Nano-structure design

It is a rough journey to deliver drugs, genes, or imaging agents to tumor tissues. For an esterase-responsive nanoparticle, passengers must stably sit in the nano-bus during CAPI steps and release or activate with esterase in the tumor cells. To realize the stability transition, several strategies are commonly utilized: (i) micelles or liposomes dissociation, (ii) drug–carrier or drug–drug conjugate with ester bonds, (iii) capped with esterase-responsive stopper, (iv) reverse carrier charge, and (v) activate inactive drug or imaging reagent. Table 3 showed the advantage and disadvantage of each strategy. We should point out that it would be impractical to expect one strategy is better than any other else. It is important that choose the suitable method for the application.

**Table 3.** Strategies for esterase nanoparticles design.

Strategies	Advantages	Disadvantages
Micelles or liposomes dissociation	Simple preparation method; Low cost	Premature drug release
Drug–carrier or drug–drug conjugate	No burst drug release; Extremely high drug loading for drug–drug conjugate	Low drug loading for drug–carrier conjugate; Few available drugs for drug–drug conjugate
Capped with esterase-responsive stopper	High drug loading capacity; Easy functionalization	Poor biodegradability
Reverse carrier charge	Strengthened endosomal escape ability	Narrow application range (for gene or charged drug mainly)
Activate inactive drug or imaging reagent	Accurate location	Narrow application range (mainly for fluorescent imaging)

## 4. Application of esterase-responsive nanoparticles in tumor treatment

### 4.1. Drug delivery

With conventional chemotherapeutics, anticancer drugs could kill tumor cells, however, unfortunately along with normal tissue cells. The biggest problem is that the conventional drug lacks the ability to distinguish tumor cells and normal cells. Esterase-responsive nanoparticles could be sensitive for tumor cells due to the overexpressed esterase in tumor cells. For a drug delivery esterase-responsive nanoparticle, strategy (i), (ii), and (iii) are most commonly used.

#### 4.1.1. Micellar drug delivery

Micellar nanoparticles, mainly consist of amphiphilic polymers and hydrophobic drugs, have become the most popular vehicle for drug delivery. Micellar nanoparticles are stable during the delivery journey, and then disassembled after swallowed into tumor cells. Biocompatible polyesters, such as PCL, polylactic acid (PLA) or poly (lactic-*co*-glycolic acid) (PLGA), are commonly used for providing hydrophobic cores. These polyesters could be hydrolyzed with esterase, and thus release drugs from micelles. In all the amphiphilic polymeric carriers, PEG-block-polyesters have been developed for antitumor drug delivery and demonstrated high performance in clinical treatment. For example, monomethoxy PEG-block-poly(d,l-lactide) (mPEG-b-PLA) has been used as carrier in a commercial micelle medicine, Genexol-PM (Lee et al., 2008). This micelle medicine is expected to treat breast cancer and non-small cell lung cancer. A great many kinds of hydrophobic anticancer drugs have been delivered by PEG-block-polyesters carriers in preclinical tests. Hydrophobic polyester cores of micelles provide a natural carrier environment for hydrophobic drugs. For example, Eawsakul et al. (2017) proposed the preparation of AG50 (one of the modified andrographolide, an anticancer drug) in two types of polymer micelles (PEG-b-PCL and PEG-b-PLA) by thin film ultrasonication. These micelles range in size from 10 to 100 nm, and the solubility of AG50 is increased up to 900 times compared to free AG50. Release studies indicated that the AG50 release from polymer micelles in PBS (pH 7.4) was much slower compared to the presence of esterase (porcine esterase). The encapsulation of AG50 in polymer micelles maintains a cytotoxic effect on cancer cells. Yadav et al. designed a PEG-PLGA deliver system for 5-fluorouracil (5-FU) (Yadav et al., 2010). PEG-PLGA was further conjugated to hyaluronic acid (HA) to possess CD44 targeting ability and then 5-FU was loaded. The nanoparticle system was found to be safe for intravenous administration and showed better cytotoxicity in cancer cells than pure 5-FU. Some other PEG micelles have also been synthesized for esterase-responsive drug delivery. Sahu et al. (2008) synthesized a novel polymeric amphiphile using methoxy poly (ethylene glycol) (mPEG) as the hydrophilic moiety and poly palmitic acid as the hydrophobic segment. Curcumin is loaded to the core of the nanocarrier and demonstrated good enzyme-triggered release *in vitro*. Encapsulation of the highly hydrophobic compound curcumin in the nanocarrier makes the drug readily soluble in water, and the ester bond can be broken by esterase in the body environment to release the curcumin. It makes drug release more efficient and makes intravenous administration possible. The drug-loaded micelle nanoparticles showed good stability under physiological conditions (pH 7.4), simulated gastric fluid (pH 1.2), and simulated intestinal fluid (pH 6.8).

**Figure 2.**
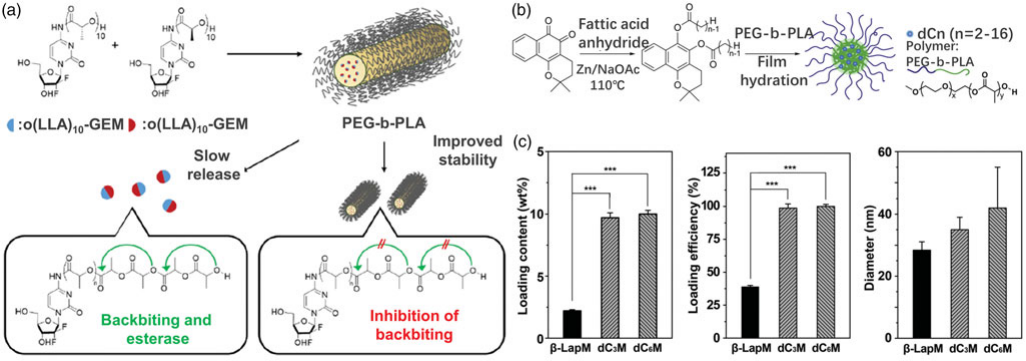
(a) OLA-modified GEM loaded in PEG-PLA micelles (Tam et al., 2018). Copyright 2018, American Chemical Society. (b) Modified β-lapachone prodrug loaded in PEG-PLA micelles. (c) Loading content, loading efficiency, and diameter of modified β-lapachone in PEG-PLA micelles (Ma et al., 2015). Copyright 2015, Elsevier. Reprinted with permission.

For some anticancer drugs, they could not be loaded in micelle core due to their incompatibility with polyesters. In that case, modification for anticancer drugs has been performed to overcome this challenge. For instance, Tam et al. (2018) reported a series of stereo complex prodrugs of oligo lactic acid-gemcitabine (OLA-GEM) for stable incorporation in PEG-b-PLA micelles. After complexation with OLA, GEM has much lower metabolism level in plasma and release drugs in tumor cells hydrolyzed by esterase. The micellular stability can be also improved through the formation of complex (Figure 2(a)). Ma et al. (2015) reported the development and preclinical evaluation of β-lapachone prodrug, β-lapachone diester derivatives, encapsulated by biodegradable PEG-b-PLA micelles (Figure 2(b)). The diester derivatives of β-lapachone showed higher drug loading density in the PEG-b-PLA micelles than the parent drug, due to crystallization of β-lapachone (Figure 2(c)). After esterase treatment, the micelle-delivered β-lapachone prodrugs were released and converted to β-lapachone. Feng et al. (2013) utilized 2-bromo hexadecanoic acid to enhance drug loading of docetaxel. The lipid conjugate of docetaxel was filled in lipid nanoparticles with high entrapment efficiency and long retention in blood. The drug did not release until entering tumor cells with high intracellular esterase concentration.

Another solution is the modification of polyesters core. Surnar et al. (2014) developed a block copolymer carrier for cisplatin delivery whose PCL backbone modified with carboxylic acid functional groups enable it to be bonded to drugs. The drug conjugate is relatively stable in saline and PBS with less cleavage, and in the presence of esterase, the biodegradable PCL ester bond cleaves instantaneously and releases all cargo (100% of the drug). Next, Surnar et al. (2015) further studied the biodegradable diblock copolymer core-shell nanoparticle module *in vivo*. In PBS and saline solution, drug stability increased with increasing PEG shell protective layer. The copolymer-carrier biodegradable aliphatic PCL ester backbone shields the cisplatin core from GSH effects, and rupture occurs only when the intracellular compartment is exposed to a higher concentration of esterase under the same conditions.

**Figure 3.**
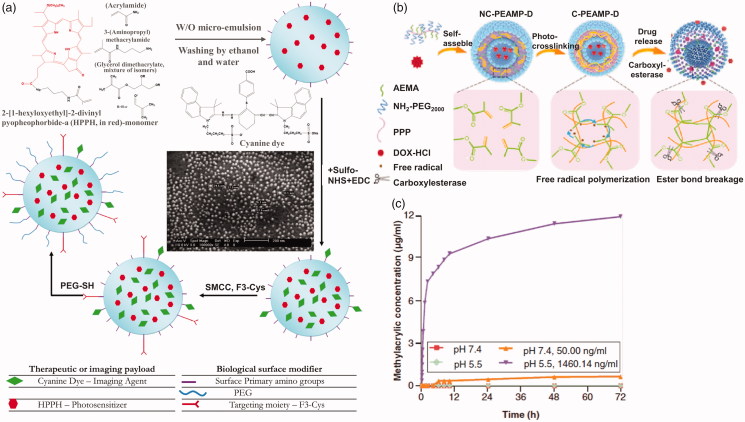
(a) A see and treat micelles nano-drug delivery crosslinked by glycerol dimethacrylate (Wang et al., 2012). Copyright 2012, American Chemical Society. (b) Photo crosslinking and esterase-responsive drug release of polymersome. (c) The methacrylic acid concentration curves at pH 7.4 and pH 5.5 without esterase or with esterase (Fu & Qiu, 2018). Copyright 2018, Future Medicine Ltd. Reprinted with permission.

**Figure 4.**
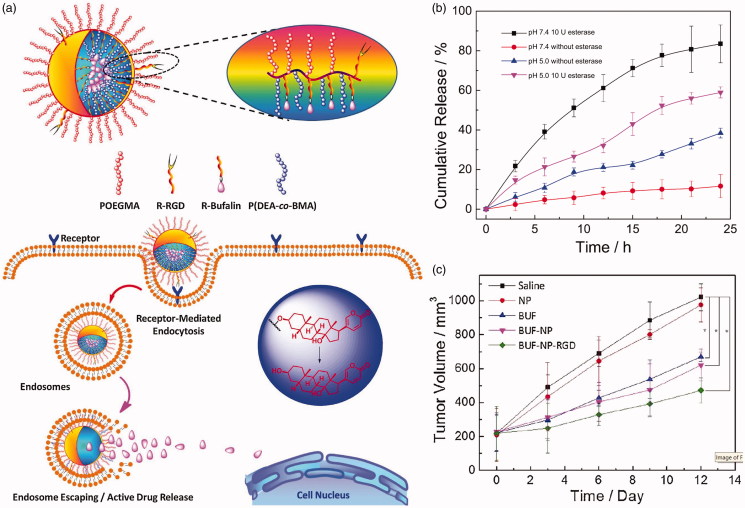
(a) Brush type polymeric prodrug covalently linked with BUF. (b) *In vitro* BUF release profiles from BUF-NP-RGD at different conditions. (c) Tumor volume changes after 12 days of intravenous injection (Shi et al., 2018). Copyright 2018, Elsevier. Reprinted with permission.

Although possessing several advantages, micellar nanoparticles suffer the premature drug release problem. A feasible solution is to crosslink the micelle core to prevent premature drug release. Crosslinkers containing ester bonds or forming ester bonds also give micelles esterase-responsive ability (Figure 3). These kinds of micelles would be de-crosslinked with hydrolysis reaction with esterase catalysis, and subsequently release drugs. Wang et al. (2012) prepared a multifunctional nanocarrier dependent on biodegradable polyacrylamide nanoparticles. Primary amino groups contained monomer, photosensitizer contained monomer and biodegradable crosslinkers were added during nanoparticles polymerization while fluorescent imaging agents, PEG and tumor-targeting ligands are conjugated to the surface of the nanoparticles (Figure 4(a)). Accelerated biodegradation studies with NaOH or pig liver esterase showed that the hydrogel polymer matrix chain collapsed within a few days. PDT and fluorescent imaging are also realized by this NP. Fu & Qiu (2018) designed a photo-crosslinked polymersome containing esterase sensitive bonds. 2-aminoethyl methacrylate on polyphosphazene backbone was polymerized after UV irradiation (Figure 4(b)). The polymersome was filled with doxorubicin (DOX) and the accelerated release occurred responsively to the esterase at high concentration in cancer cells (Figure 4(c)). The crosslinked polymersome showed 3.1-fold prolonged halftime of drug compared with non-crosslinked polymersome. However, crosslinked micelles could only reduce the premature release problem partially. To overcome the premature release problem, drug-carriers or drug–drug covalently conjugates have been employed.

#### 4.1.2. Prodrug

Another frequently used strategy is drug–carrier conjugate, which is commonly called polymeric prodrug. Drugs and polymeric carriers are linked by ester bonds to form nanoparticles, and prodrugs are often effective tools to overcome the deficiencies associated with drug preparation and delivery. The polymeric prodrugs commonly have no burst release problems due to the ester bonds are stable in blood circulation. Once taken by tumor cells, the prodrugs are divided by esterase to release drugs. Commonly, hydrophobic anticancer drug is linked to the micellular core. For linking drugs to polymeric carriers, several strategies are introduced. One of the most commonly used methods is that drugs are conjugated to a polymer by an ester bond. For example, Zhou et al. (2016) synthesized biodegradable poly (ethylene glycol-anhydride). This polyester-based polymer has a plurality of carboxylic acid groups and can be used as a good pharmaceutical carrier. Two anticancer drugs, camptothecin (CPT) and DOX, were selectively conjugated onto the polymer carrier to study their release properties and anticancer activity *in vitro*. These polymer–drug conjugates are substantially stable in the absence of esterase, but are rapidly hydrolyzed by reaction with intracellular esterase to release the drug in the cells. Esterase promotes hydrolysis of the polymer–drug conjugate and drug release. Shi et al. (2018) developed a brush-type polymer that is covalently linked with an antitumor medicine bufalin (BUF) (Figure 4(a)). This polymer could be assembled into nanoparticles (BUF-NP-RGD) in aqueous condition and exhibited controlled drug release in the presence of esterase (Figure 4(b)). The BUF-NP-RGD can more effectively induce tumor cells apoptosis *in vivo* (Figure 4(c)). Prior to this, a similar brush-type polymer was prepared for breast cancer (Jia et al., 2016). Carbohydrate polymers and their derivatives were often used to form prodrugs due to a large quantity of hydroxyl groups or carboxyl groups on their backbones. HA (Su et al., 2018), alginate (Noverraz et al., 2018), heparin (Li et al., 2014), etc. have been modified with anticancer drugs via ester bonds.

**Figure 5.**
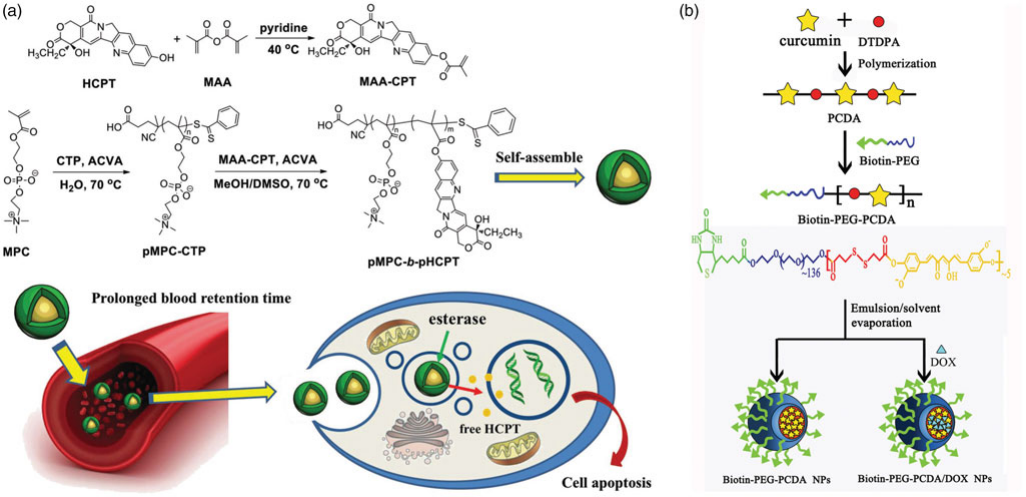
(a) Polymerization of MAA-CPT and MPC by two-step RAFT method (Chen et al., 2017). Copyright 2017, Wiley. (b) Preparation of biotin-PEG-PCDA and DOX-loaded biotin-PEG-PCDA NPs (Lv et al., 2015). Copyright 2015, Wiley. Reprinted with permission.

Another strategy is that drugs are linked to monomer molecules, such as methacrylic acid, or polymerizable themselves, and then obtained prodrug by polymerization. Chen et al. (2017) introduced methacrylic acid to HCPT to form a polymerizable monomer. Subsequently, the drug-containing monomer (MAA-CPT) block-polymerized by a two-step RAFT method with 2-methylacryloyloxyethyl phosphorylcholine (MPC), a zwitterionic monomer with antifouling property in plasma (Figure 5(a)). This block polymer can self-assemble into micelles and release HCPT under high esterase concentration in tumor cells. Tucker et al. (2015) linked *N*-(2-hydroxypropyl) methacrylamide (HPMA) and methotrexate (MTX) by an ester bond, and the copolymerized with HPMA and a divinyl crosslinker, dimethacrylate glycol, by using RAFT method. The drugs are loaded in the core of poly-HPMA star polymers. The HPMA–MTX conjugates would release drugs under esterase catalysis. Condensation polymerization has been also employed to prepare polymeric prodrugs. Curcumin, possess two phenol hydroxy group, can be polymerized with dicarboxylic acid compound (Tang et al., 2010). Lv et al. (2015) used curcumin and dithiodi-propionic acid to form a polymeric prodrug (Figure 5(b)). The curcumin prodrug subsequently linked to a biotin-PEG to prepare an amphiphilic polymer. DOX was also loaded along with the amphiphilic polymeric micelle produced. This nanomedicine offers high curcumin release rate, companied with DOX, under intracellular esterase condition.

**Figure 6.**
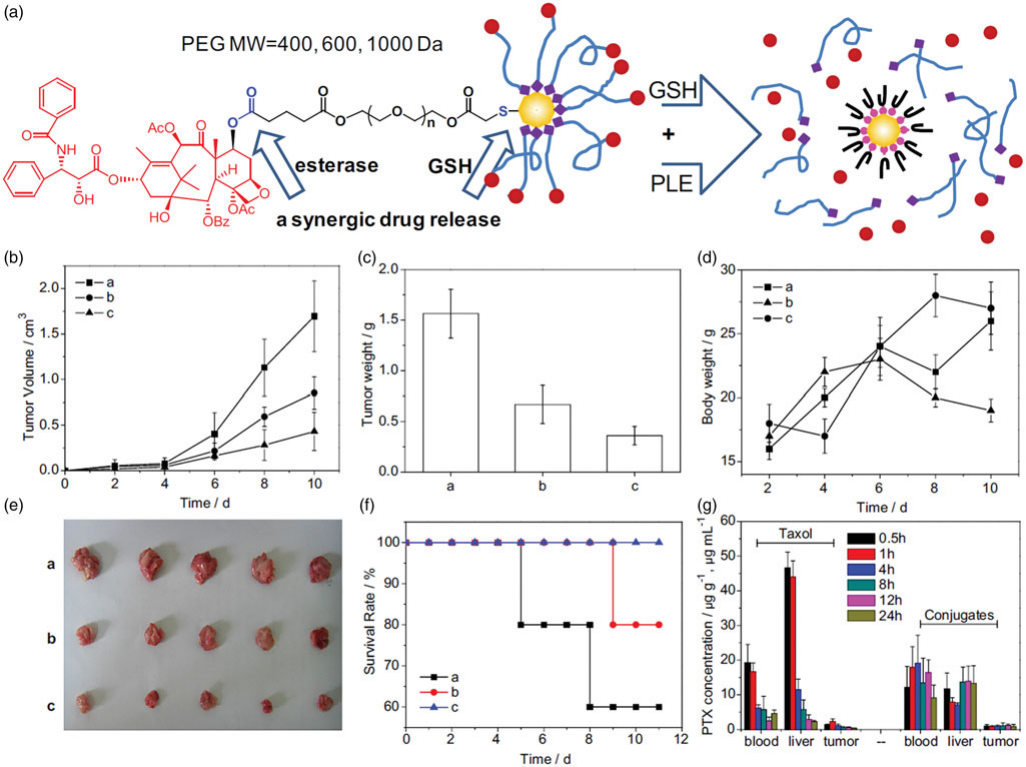
(a) Schematic illustration of the chemical structure of PTX-PEG@GNP conjugates. (b–g) Tumor volume, tumor weight, body weight, images of tumor tissues, survival rates, and PTX concentration in Heps tumor xenograft ICR mouse models (Ding et al., 2013). Copyright 2013, Elsevier. Reprinted with permission.

A third modality for esterase response to drug carriers is to link hydrophobic drugs to the terminal of a drug delivery vehicle. The drug remains stable *in vivo* microenvironment. When a higher esterase concentration environment is encountered, the terminator will be activated and the drug will be released. PEG-terminal drug conjugates are the most commonly used. For example, Ding et al. (2013) designed and synthesized a series of thiol-terminated PEG of which paclitaxel (PTX) was linked on the other terminal (HS-PEG-PTX). HS-PEG-PTX derivatives were further conjugated to gold nanoparticles (PTX@GNP). PTX@GNP conjugates exhibit dual stimulus-induced drug release behavior in the presence of esterase and high concentrations of GSH. The synergistic release of this conjugate results in improved performance, including long circulation due to high stability *in vivo*, targeted release of PTX in tumor cells, and increased tumor cell killing efficiency (Figure 6). Mattheolabakis et al. (2014) have designed a phospho-ibuprofen prodrug by covalently linking with PEG. The phosphor-ibuprofen could be hydrolyzed by esterase in tumor cells and reduce tumor growth rate significantly. Similar works also performed to form PEG-drug conjugate by various method (Jayant et al., 2007; Lee et al., 2010; Shen et al., 2010; Chen et al., 2014). Peptide has been another one of the most popular carriers in drug delivery. DTS-108 (Meyer-Losic et al., 2008), a hydrosoluble prodrug consist of SN-38 and a 20 amino acids peptide. SN-38 moiety is covalently linked to the peptide vehicle by an esterase-responsive linker. A phase I study has been reported and the results showed that the maximum tolerated dose of DTS-108 was 416 mg/m^2^ (Coriat et al., 2016). Some other carriers, such as lipid analog (Alam et al., 2015), poly carboxybetaine (Li et al., 2014), inorganic delivery (Cengelli et al., 2009; Zhu et al., 2018), etc., have also been utilized for prodrug delivery.

**Figure 7.**
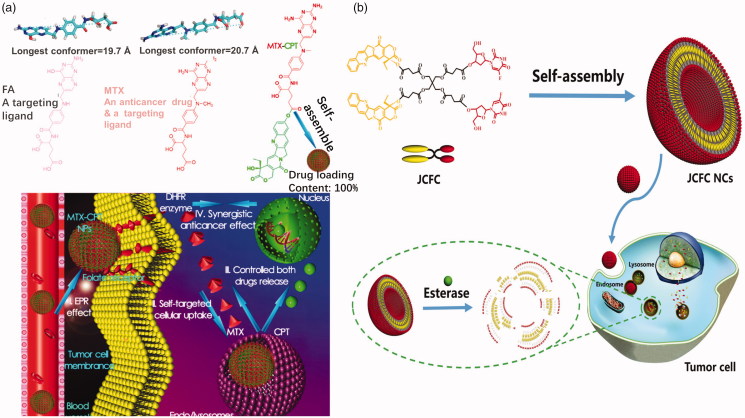
(a) Optimized molecular structures and schematic illustration of MTX-CPT (Li et al., 2017). Copyright 2017, American Chemical Society. (b) Janus drug–drug conjugates linked by pentaerythritol (Liang et al., 2017). Copyright 2017, Wiley. Reprinted with permission.

The carrier–drug conjugate strategy could reduce, even eliminate, drug burst release questions, however, drug-loading content problem has raised due to the high carrier weight ratio. To overcome the drug-loading problems, drug–drug conjugates type prodrugs have been designed. These kinds of prodrugs consist of two or more drugs which are linked by an ester bond directly or a linker with ester bond. Drug content of the drug–drug conjugate prodrugs is very high, some of which is almost 100%. Generally, the prodrug consists of a hydrophilic drug and a hydrophobic drug to form an amphiphilic molecule. The amphiphilic prodrug could assemble into micelles in aqueous solution. Li et al. (2017) adopted a combination of MTX and HCPT directly by an enzyme-cleavable ester bond to prepare an antitumor drug–drug conjugate prodrug (Figure 7(a)). This prodrug was subsequently loaded into a 1,2-distearoyl-snglycero-3-phosphoethanolamine (DSPE)–HA–MTX drug delivery (Li et al., 2018). MTX, an analog of folic acid, can be served as a tumor-specific targeting ligand as well as an anticancer drug. The ester bond between MTX and HCPT can be cleaved by esterase and acid condition in tumor cells. Sheng et al. (2017) utilized cantharidin (CTR), as the hydrophilic drug, conjugated to CPT. The CTR-CPT prodrug could be also opened by esterase in tumor cells. Obviously, a carboxyl group and a hydroxyl group must be existed on the two drug molecules, respectively, to form an esterase-responsive drug–drug conjugate directly. If not, a proper linker is needed for linking drugs. Liang et al. (2017) successfully constructed novel homogeneous liposome nanocapsules by self-assembly in aqueous solution. The capsule is synthesized from a highly symmetrical Janus CPT-fluorouridine (FUDR) conjugate (JCFC) amphiphile with a phospholipid mimetic structure (Figure 7(b)). The amphiphilic FUDR molecule is synthesized by a hydrolyzable ester bond coupled to polyvalent pentaerythritol by a protection–deprotection strategy. Due to its amphiphilic structure, JCFC can self-assemble into uniform double-layer nanocapsules with a solid molar ratio of CPT/FUDR (1:1) to achieve synergistic antitumor activity. Due to the use of the drug–drug conjugate itself as a carrier, JCFC NCs have a very high drug loading, a highly stable coded drug combination and no premature release. Due to esterase and acid hydrolysis of ester bonds in tumor cells, CPT and FUDR can be released synergistically from JCFC NCs, resulting in a higher apoptotic rate and synergistic anticancer activity than the individual free drug and CPT and FUDR mixture. Furthermore, they utilized a combination of JCFC, 1,1′-dioctadecyl-3,3,3′,3′-tetramethylindotricarbocyanine iodide (DiR, a near-infrared light absorber), and DSPE-PEG to prepare a nanomedicine for chemophotothermal therapy (Gao et al., 2018). Obviously, the drug–drug conjugates prodrugs possess extremely high drug loading content, however, only a few of drugs can be used to form these kinds of prodrugs because the structure of drug molecules is with exact requirement, such as hydrophilicity or hydrophobicity of drugs, carboxyl groups and hydroxyl groups on drug molecules. Moreover, the two or more drugs used must be with synergistic treatment effect. For these reasons, the application of drug–drug conjugate prodrugs is restricted clinically.

#### 4.1.3. Capped with esterase stopper

**Figure 8.**
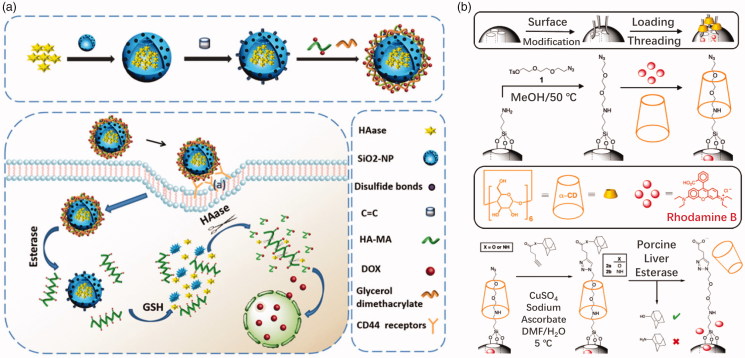
(a) Crosslinked HA covered degradable MSN delivery (Chen et al., 2018). Copyright 2018, The Royal Society of Chemistry. (b) A ‘snap-top’ covered MSN drug delivery (Patel et al., 2008). Copyright 2008, American Chemical Society. Reprinted with permission.

Mesoporous nano-materials, especially mesoporous silica nano-materials (MSNs), have been extensively studied due to their vast advantages. In general, drugs are loaded in mesopores of MSNs and then capped by a stopper, commonly a polymeric stopper. Once entered tumor cells, the stopper would be split from MSNs to release drugs under tumor microenvironment, such as extremely high esterase concentration. Polyesters are commonly used to cap the MSNs and PCL is also one of the most favorite polyesters. Chen et al. (2014) prepared a PCL capped MSN drug carrier. Polyacrylic acid (PAA) is further capped on the surface of MSN carrier. DOX loaded in the MSN carrier would only release after cleavage of PAA (at low pH) and digestion of PCL (under high esterase concentration) in a stepwise method. Fernando et al. (2015) reported the development of esterase and pH-responsive poly (β-aminoester)-terminated MSNs (POL-MSN) integrated systems as controlled drug delivery vehicles. Modification of the polymer backbone with ester functional groups facilitates esterase cleavage of the polymer. They combine the pH-sensitive advantages of borate with the simple advantages of synthetic poly (β-amino ester) synthesis. In tumor therapy, polymer-terminated MSNs are more effective than single agents which are responsive in providing antitumor drugs. In tumor cells microenvironment, it responds to the environment of low pH and high concentration of esterase, releasing the drugs it carries. Bernardos et al. (2012) synthesized a kind of novel polyesters, bearing side-chain azobenzene moieties, for capping MSNs. The bulky azo groups on the polyesters generate holes in the polymeric backbone, resulting in acceleration of hydrolysis by esterase. However, poor biodegradability has hampered their clinical treatment. Degradable silica hybrids have been reported (Hao et al., 2015) and the esterase-responsive MSNs drug delivery could be designed based on these materials to solve the degradable problem. Chen et al. (2018) utilized GSH degradable silica nanoparticles containing disulfide bonds for cancer drug delivery. Polyester-hyaluronic acid–DOX corona coated the silica nanoparticles which were filled with hyaluronidase (Figure 8(a)). Polyester on hyaluronic acid backbones was hydrolyzed under tumorous esterase and hyaluronic acid–DOX conjugates were further released free DOX as long as hyaluronidase is present, which was released from silica core in the cytoplasm (rich in GSH). The drug was not released until incubation of the nanomedicine with both esterase and GSH.

Another strategy is that MSNs are covalently linked to polymers with ester bonds. Agostini et al. (2012) linked MSNs and PEG via an ester bond to synthesize a nanoscopic gated material for cargoes controlled release. The ester linkages would be hydrolyzed under enzyme catalyst and PEG shell would fall off accompanied with drug release. Patel et al. (2008) designed a so-called ‘snap-top’ covered MSN drug delivery. Cyclodextrin was tethered on the surface of MSN that modified with an azide group. Subsequently, alkyne ester-linked adamantyl stopper was capped onto the MSN by click reaction. The adamantyl stopper was hydrolyzed by esterase to detether cyclodextrin and release cargo of molecules (Figure 8(b)). MSNs hold a great potential as drug carriers for tumor therapy due to their high drug loading property, ease of surface modification, good biocompatible, etc.

### 4.2. Gene deliveries

**Figure 9.**
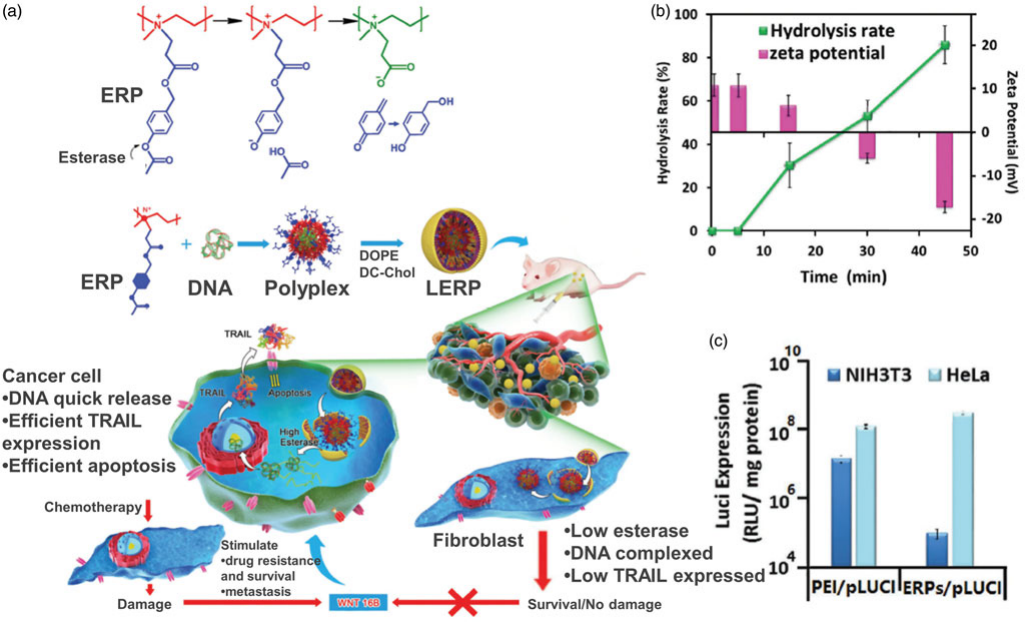
(a) Esterase-responsive charge reversal gene delivery. (b) Hydrolysis rate and zeta potential of ERP gene delivery. (c) Luci expression of ERP in NIH3T3 and HeLa cell lines (Qiu et al., 2016). Copyright 2016, Wiley. Reprinted with permission.

Gene therapies for tumor treatment have been conducted clinically and showed good tolerability. However, naked genes are often cleared by systemic circulation quickly resulting in a poor clinal treatment efficiency. Cationic carriers have been extensively explored as non-viral gene delivery due to their nonimmonogenity and safe nature (Zhou et al., 2017). These cationic carrier materials can carry gene plasmids, which are negatively charged, and form an effective protection. After entering tumor cells, the cationic carrier should be turned to neutral or negatively charged to release gene plasmids. This procedure is called ‘charge reversal.’ Esterase, one of the overexpressed enzymes in tumor cells, has been utilized to realize the charge reversal transition. Shen’s group did a lot of work in this area. They designed and synthesized a cationic polymer, quaternized PEI by *p*-acetoxybenzyl acrylate, that can respond to intracellular esterase and charge reversal (Qiu et al., 2016) (Figure 9(a)). Esterase-responsive polymer (ERP) can effectively encapsulate compressed DNA in the cationic state to form stable nanoparticles with a particle size of about 80 nm and a potential of +15 mV. After entering tumor cells, under the action of intracellular esterase, the acetyl phenolic ester of ERP will be rapidly hydrolyzed, triggering the release of the *p*-hydroxyl benzyl alcohol reaction, directly generating carboxylate and forming a neutral poly ion pair with the amino group, realizing the charge reversal. The polymer’s potential changes from positive to negative causing the polymer to lose its interaction with DNA’s and rapidly dissociate the complex and release the DNA (Figure 9(b)). The ERP carrying Luci gene plasmid effectively expressed in Hela cells but not NIH3T3 cells, one of fibroblasts cells, to secret WNT16B, reducing the possibility of tumor metastasis (Figure 9(c)). Furthermore, they replaced PEI with poly 2-(*N*,*N*-diethylamino)ethyl acrylate to synthesis a charge reversal gene delivery (Qiu et al., 2018). Both carriers delivered TRAIL gene plasmid effectively and showed better treatment efficiency than two first-line antitumor drugs, CPT-11 and PTX. Zhang et al. (2017) designed a series of biodegradable lipid-like compounds, which contain ester groups, for the delivery of mRNA-encoding Cas9. The vectors degradable rate can be controlled by changing the functional groups on the ester chains. The lipid-like carrier would expose carboxyl groups accompanied with esterase catalysis hydrolysis, leading to the positive charge turning to neutral.

**Figure 10.**
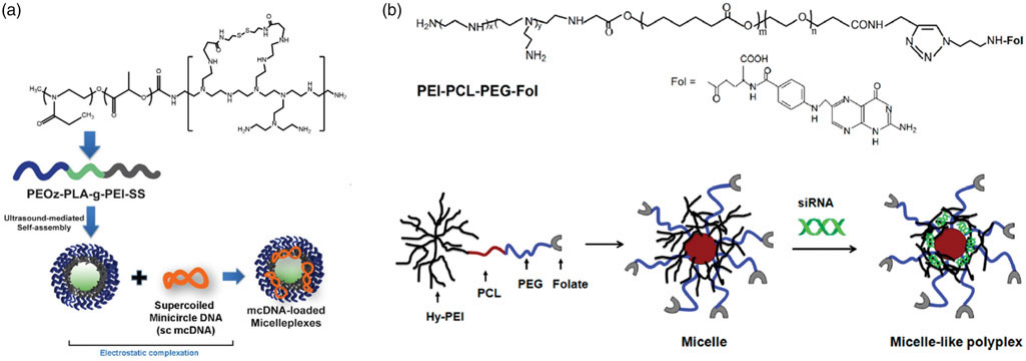
(a) Schematics of PEOz-PLA-PEI micelles for DNA delivery (Gaspar et al., 2015). Copyright 2015, Elsevier. (b) Chemical structure of PEI − PCL − PEG − Fol and schematic illustration of the micelle-like polyplex formation (Liu et al., 2016). Copyright 2015, American Chemical Society. Reprinted with permission.

These kinds of polymer–gene plasmid complex, which is always called polyplex, are less stable than polymer micellular gene carriers (Yousefpour and Yari, 2017). Just as drug carriers, PEG-polyesters are also favored for gene delivery due to their esterase responsiveness. Kanazawa et al. (2012) utilized mPEG-PCL nanomicelles to deliver siRNA. To facilitate intracellular uptake, TAT, a cell penetrating peptide, is linked to mPEG-PCL by a disulfide bond. Pure PEG-polyesters block polymer has limited capacity of loading gene plasmid. In that case, PEG-polyesters-block/graft-polycation type co-polymers are used for gene delivery, where PEI is the most popular cationic polymer. Shi et al. (2012) designed a series of triblock copolymer, PEG–poly ε-caprolactone–polyethylenimine (mPEG–PCL-g–PEI), for co-delivery DNA plasmid and drugs. These copolymers could self-assemble into micelles with positive charge. Further, the copolymers were studied in detail, including *in vitro* drug release behavior, body distribution as well as blood compatibility (Shi et al., 2014). The gene, along with DOX, loaded micelles showed extraordinary properties in tumor treatment. Liu et al. (2016) utilized folate modified PEI-PCL-PEG triblock polymer to deliver siRNA plasmid (Figure 10(b)). The folate-conjugated micelles showed tumor targeting ability. PLA is also used as hydrophobic core of gene micellular vectors (Qian et al., 2014; Gaspar et al., 2015; Ni et al., 2018). Some other cationic polymers are also blocked/grafted to PEG-polyesters backbone. Zhu et al. (2008) synthesized a series of PEG-b-polycation-co-(HEMA-g-PCL) by RAFT method. The polycation is poly *N*-[3-(dimethylamino) propyl] methacryl amide. The amphiphilic copolymer could also load gene and drugs for tumor treatment. However, the cationic micelle gene carriers above lack charge reversal property. In that case, cationic micelles with esterase-responsive charge reversal property would meet the requirement of gene delivery.

### 4.3. PDT and PTT

PDTs and PTTs have been intensively studied for the treatment of various tumors. Both PDT and PTT are mild and noninvasive therapies compared to conventional methods, such as surgery, chemotherapy, and radiation therapy. The principle of PDT and PTT is that photosensitizers generate reactive oxygen or heat, respectively, after the absorption of light irradiation. Fluorescence light sometimes accompanied with PDT or PTT of photosensitizer and could be used for tumor imaging. The fluorescent imaging for tumor tissues is discussed in the next section.

**Figure 11.**
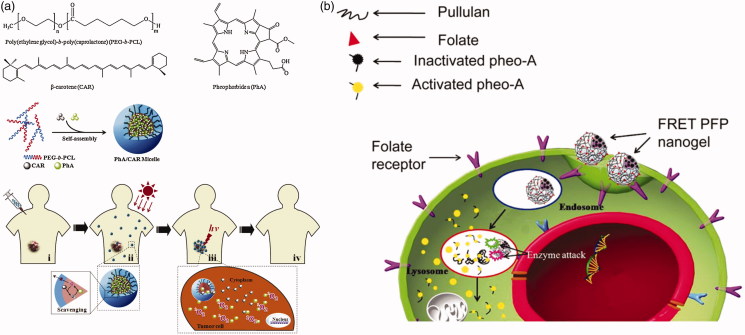
(a) ^1^O_2_ scavenging in PhA/CAR micelles and photokilling activity in the intracellular environment (Li et al., 2014). Copyright 2014, Elsevier. (b) Self-quenching of photosensitizers during blood circulation and photoactivity is restored under esterase catalysis (Bae & Na, 2010). Copyright 2010, Elsevier. Reprinted with permission.

To deliver photosensitizer to tumor cells, micellular vectors are also commonly used. Polyesters, such as PCL, PLA, are chosen to be the core of micelles. Several polyesters-based micelles are used for photosensitizer delivery (Wu et al., 2010; Dong et al., 2016; Jeong et al., 2016; Fan et al., 2017; Ren et al., 2017). Unlike drug or gene delivery, photosensitizer release is not a prerequisite for PDT or PTT action (Liang et al., 2011). However, some studies applied delivery for esterase activating photosensitizer. Li et al. (2014) prepared a dual-loaded PEG-PCL micelle with pheophorbide A (PhA) as a photosensitizer and beta-carotene (CAR) as a singlet oxygen scavenger (Figure 11(a)). The singlet oxygen generated by photosensitizer would be eliminated by CAR in the micelles. After the micelles disassemble under high esterase concentration in tumor cells, the singlet oxygen would kill tumor cells because of the termination of the scavenging reactions. Bae & Na (2010) designed a nanogel with self-quenchable photoactivity by conjugated photosensitizer on pullulan backbone with an ester bond (Figure 11(b)). The photosensitizer did not show PDT efficiency due to a similar effect of fluorescence resonance energy transfer effect. Both fluorescent identity and PDT efficiency of the nanogel are increased after hydrolyzed by esterase.

As mentioned above, photosensitizer does not need to be released to tumor cells, not much studies put emphasis on esterase-responsive release for photosensitizer directly. Protoporphyrin IX (PpIX), a PDT agent for tumor treatment, could be synthesized intercellularly from 5-aminolevulinic acid (ALA) precursor. The ALA, however, is hydrophilic which results in poor ability to enter cells. Esters of ALA are ideal substitutes for ALA due to their increasing internalization efficiency with high lipophilicity. Once enter tumor cells, esters of ALA would be hydrolyzed under high esterase concentration. For examples, hexyl ester of ALA has been used for tumor PDT (Bigelow et al., 2001; Perotti et al., 2002). The hexyl ester of ALA has better cellular penetration ability and release ALA in tumor cells or incubated with esterase. A series of aliphatic esters of ALA are synthesized (Bigelow et al., 2001; Tunstall et al., 2002; Perotti et al., 2004; Di Venosa et al., 2006) for PDT. Some other esters also have been designed for replacing ALA. Berger et al. (2000) synthesized a series of OEG or amino acid esters with ALA, which could produce high PpIX concentration under intracellular esterase. Vallinayagam et al. (2008) utilized several monosaccharides to conjugate ALA with an ester linkage. Such esters of ALA could enhance lipophilicity of ALA as well as compatibility of cell membranes or micelle cores, which could be possible further delivered by amphiphilic carriers, such as PEG-PCL or PEG-PLA. Carrier–drug conjugate strategy is also suitable for ALA delivery. Babic et al. (2018) designed a squalene ester of ALA, which could be self-assembled into nanoparticles. The nanoparticles were effectively inducing PpIX production in PC3 and U87MG types cancer cells. Zhou et al. (2018) synthesize a series of dendrimers to co-deliver ALA and hydroxypyridinone (HPO). Both drugs, which act synergistically to amplify *in vitro* PpIX levels, are conjugated to dendrimer by ester bonds. Another dendrimer prepared by Casas et al. (2009) has also been used for ALA delivery. From the above, esterase activating and prodrug strategies are often used for PDT or PTT and micelles could be potential candidates in the future.

### 4.4. Tumor imaging

**Figure 12.**
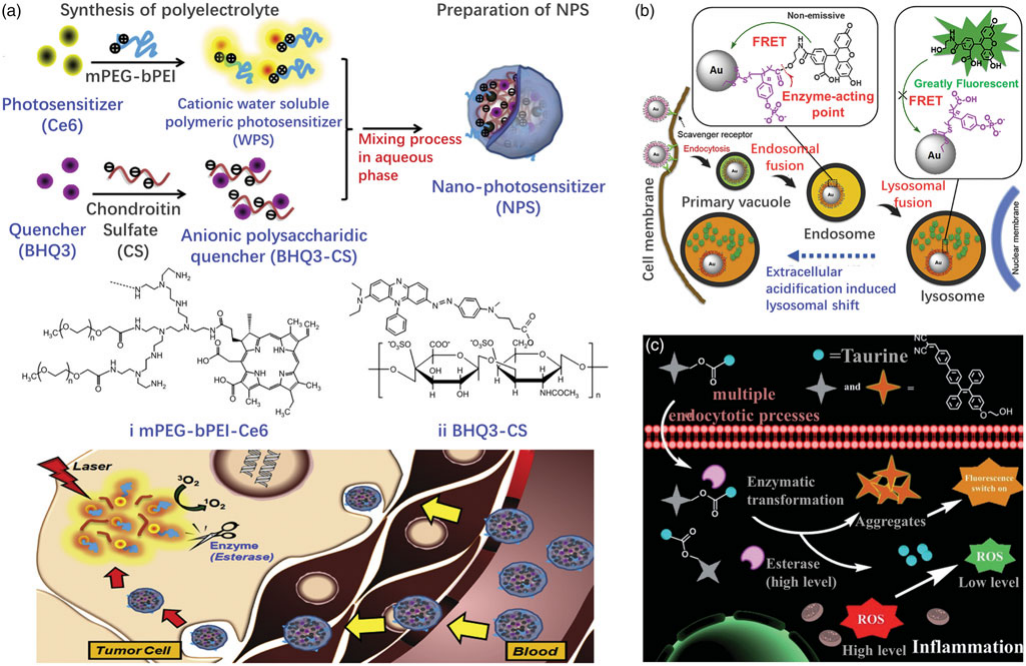
(a) Schematic of NPS diagnosis ability originating from enzymatic triggering (Park et al., 2011). Copyright 2011, Elsevier. (b) Esterase-switched fluorescence ‘off-on’ nanoprobe to illuminate the lysosome (Fan et al., 2014). Copyright 2014, Elsevier. (c) Schematic illustration of cellular uptake for the system and the subsequent esterase-activated fluorescence switching on (Wang et al., 2017). Copyright 2017, The Royal Society of Chemistry. Reprinted with permission.

Esterase-responsive nanoparticles are also often used to activate inactive imaging agents for the purpose of treating tumors. One such approach is to attach a fluorescein or photosensitizer to a quencher molecule in esterase sensitive carriers. A nanophotosensitive agent (NPS) was prepared, for example, by Park et al. (2011) using a polyelectrolyte complex between PEG-polyethyleneimine-Ce6 conjugate (PEG-PEI-Ce6) and Black Hole Quencher-3 chondroitin sulfate conjugate (BHQ-3-CS) (Figure 12(a)). The photoquenching of NPS depends on the weight ratio of BHQ-3-CS/PEG-PEI-Ce6. NPS apparently loses photoactivity through the intermolecular FRET effect in the aqueous phase and the enzymatic degradation of BHQ-3-CS after esterase treatment restored the photoactivity of NPS. When NPS was injected subcutaneously in the tumor and normal areas of mice carrying HCT-116 tumors, the fluorescent signal in the tumor rapidly increased compared to the normal area due to enzymatically triggered NPS dissociation *in vivo*. Fan et al. (2014) designed an esterase-responsive nanoparticle with 16 nm spherical gold nanoparticles as the core and enzyme-responsive oligomer, that is fluorescein-conjugated oligo (4-vinyl-phenyl phosphate), as its outer shell (Figure 12(b)). Nanoparticles are nonfluorescent due to the quenching of gold core. Upon entry into the tumor cells, the nanoparticles are rapidly internalized by scavenger receptor-mediated endocytosis and significantly reorganized into lysosomes. A high concentration of esterase in the lysosome hydrolyzes the nanoparticles and releases the fluorescein molecules, illuminating the lysosome. Imaging of lysosomal displacement can be applied to cancer diagnosis due to the large association of anterograde transport with tumor-associated extracellular acidification and growth factors. Wang et al. (2017) designed and synthesized a fluorescent illumination system, consisted of hydrophilic taurine and a kind of aggregation-induced emission (AIE) fluorophore, with enhanced cellular uptake ability (Figure 12(c)). The system has a large Stokes shift, low cytotoxicity, and good light stability. The AIE fluorophore can be activated by an esterase that is overexpressed. In addition, the released hydrophilic taurine can be used to scavenge the intracellular ROS. The drug–fluorophore nanoparticle showed great potential for visualized therapy. Ji et al. (2017) linked an ester masked coumaric acids molecule to gold nanorod surface. The masked coumaric acid lost fluorescent activity and can be enzyme-hydrolyzed to form fluorescent coumarin for tumor imaging. Some drugs also possess fluorescence imaging ability themselves. Li et al. (2017) selected MTX and HCPT to conjugate via an ester bond. Due to the amphiphilic nature, the MTX-HCPT conjugate can self-assemble into MTX-HCPT nanoparticles (MTX-HCPT NPs) in aqueous solution. They later developed an amphiphilic polymer prodrug DSPE-HA-MTX-HA self-assembling nanoparticles for bimolecular targeted delivery of HCPT-MTX drug–drug conjugates to CD44/folate receptors (Li et al., 2018). HCPT, a chemotherapeutic drug with fluorescent property, inactivated its fluorescence linked to MTX. An obvious esterase triggered ‘turn-on’ effect was exhibited along with CPT release from MTX-CPT NPs. Esterification of the imaging agent is helpful to enter cells through the lipophilic membrane and activate in tumor cells, resulting in more accurate imaging. Some of the esterase-activating fluorescent probes have also the potential for tumor imaging in the future (Kamiya et al., 2007; Thompson et al., 2012; Singh et al., 2013).

## 5. Perspectives

Esterase-responsive nanoparticles have the ability to target tumor cells avoiding unwanted side effects in normal tissues. Compared to the commonly used antitumor nanodrugs on the market, they reduce the release of antitumor drugs in normal cells by the selectivity of high concentrations of esterase in tumor cells. However, the esterase response is only one of a variety of stimuli–response methods at this stage, and it should not be used as a clinical means. The most significant reason is that esterase is also overexpressed in other pathological cells, such as inflammatory cells, resulting in off-target problems. Another disadvantage is that esterase activity in tumor cells is different from person to person (Niu et al., 2012). In that case, some nanoparticles can respond to multi-stimuli simultaneously, such as GSH/esterase (Lv et al., 2015), pH/esterase (Fernando et al., 2015; Saxena & Jayakannan, 2016), heat/esterase (Kashyap et al., 2016; Aluri et al., 2018), etc. A multi-stimulus responsive drug carrier can simultaneously respond to different stimulating factors in tumor tissues and cells, not only can promote the uptake of drug conjugates by cells, but also promote the releasing of drugs in tumors, allowing the drug to function better in target cells. At present, this technical experiment only enters the preclinical stage, and its anticancer effect in the body remains to be evaluated. The esterase nanoparticle drug carrier still needs further optimization.
